# Supplementary material for: Magnetized fast isochoric laser heating for efficient creation of ultra-high-energy-density states
Source: Nat Commun. 2018 Sep 26;9:3937. doi: 10.1038/s41467-018-06173-6 (PMC6158241; doi:10.1038/s41467-018-06173-6)
Supplement: Supplementary file 1 — Supplementary Information [file 41467_2018_6173_MOESM1_ESM.pdf]

Supplementary Information for

Magnetized Fast Isochoric Laser Heating for Efficient Creation of Ultra-High-Energy-Density States

Shohei Sakata[1], Seungho Lee[1], Hiroki Morita[1], Tomoyuki Johzaki[2], Hiroshi Sawada[1,3], Yuki Iwasa[1], Kazuki Matsuo[1], King Fai Farley Law[1], Akira Yao[1], Masayasu Hata[1], Atsushi Sunahara[4], Sadaoki Kojima[1], Yuki Abe[1], Hidetaka Kishimoto[1], Aneez Syuhada[1], Takashi Shiroto[5], Alessio Morace[1], Akifumi Yogo[1], Natsumi Iwata[1], Mitsuo Nakai[1], Hitoshi Sakagami[6], Tetsuo Ozaki[6], Kohei Yamanoi[1], Takayoshi Norimatsu[1], Yoshiki Nakata[1], Shigeki Tokita[1], Noriaki Miyanaga[1], Junji Kawanaka[1], Hiroyuki Shiraga[1], Kunioki Mima[1,7], Hiroaki Nishimura[1], Mathieu Bailly-Grandvaux[8], Joao Jorge Santos[8], Hideo Nagatomo[1], Hiroshi Azechi[1], Ryosuke Kodama[1], Yasunobu Arikawa[1], Yasuhiko Sentoku[1], & Shinsuke Fujioka[1*]

[1] *Institute of Laser Engineering, Osaka University, 2-6 Yamada-Oka, Suita, Osaka, 565-0871 Japan.*

[2] *Department of Mechanical Systems Engineering, Hiroshima University, Higashi-Hiroshima, Hiroshima, 739-8527, Japan.*

[3] *Department of Physics, University of Nevada Reno, Reno, Nevada 98557, USA.*

[4] *Institute for Laser Technology, 1-8-4 Utsubo-honmachi, Nishi-ku Osaka, Osaka, 550-0004, Japan.*

[5] *Department of Aerospace Engineering, Tohoku University, Sendai, Miyagi 980-8579, Japan.*

[6] *National Institute for Fusion Science, National Institutes of Natural Sciences, 322-6 Oroshi, Toki, Gifu, 509-5292, Japan.*

[7] *The Graduate School for the Creation of New Photonics Industries, 1955-1, Kurematsu, Nishi-ku, Hamamatsu, Shizuoka 431-1202, Japan.*

[8] *University of Bordeaux, CNRS, CEA, CELIA (Centre Lasers Intenses et Applications),*

UMR 5107, F-33405 Talence, France.

Correspondence to: sfujioka@ile.osaka-u.ac.jp

This PDF file includes:

Supplementary Discussion and Methods

Supplementary Figures 1 to 2

Supplementary Tables 1

Supplementary Discussion

Previous experimental results of magnetic field generation with laser-driven scheme

Supplementary Table 1 summarizes previous experimental results [1–5] of magnetic field generation with laser-driven coil scheme conducted on kilo-Joule class laser facilities. Current flows in the coils were evaluated from the measured magnetic field strengths with resistances and inductances that were calculated for the initial coil geometries. 200 kA-level currents were obtained with except for Ref. [3]. In Ref. [3], a plastic spacer was inserted between the capacitor-plates, a current may flow in not only the coil but also the spacer surface. This target design is completely different from the other ones.

Integrated REB transport simulation

The integrated REB transport simulation was performed by coupling two-dimensional hybrid code [6] and two-dimensional radiative magneto-hydrodynamics (MHD) code [7]. Two-dimensional radiative MHD code calculated density and magnetic field profiles of a compressed solid ball attached to a gold cone. The initial magnetic field profile is shown in Fig. 3 (b) of the main manuscript. The profiles at the maximum compression timing are shown in Supplementary Fig. 1 (a) and (b), respectively. The total energy and the wavelength of the compression laser were 1.5 kJ and 0.53 μm, respectively. The pulse shape was a Gaussian with 1.3 ns of FWHM.

The REB transport simulation was performed with the profiles shown in Supplementary Fig. 1 (a) and (b). The REB was injected at z = 65 μm (dashed white line). The half divergence angle of REB was 45 degrees. Temporal shape and spatial profile of the injected REB were a Gaussian with 1 ps duration (FWHM) and the super Gaussian with 30 μm radius (FWHM), respectively. The peak intensity of REB was 7 × 10^{18} W/cm^2. The energy distribution of REB was $f(E) \propto 0.76 \exp(-E/0.9) + 0.24 \exp(-E/5)$ [8], here E is electron kinetic energy in MeV unit. This distribution was obtained by coupling high energy x-ray spectrometer and electron energy spectrometer.

The multiplication factor of the REB-to-core energy coupling was calculated by changing initial magnetic field strength at the coil center as shown in Supplementary Fig. 1 (c). The multiplication factor is the ratio between the couplings calculated with and

without application of the external magnetic field. The blue hatching indicates the range of the experimentally obtained multiplication factor including errors that is the ratio between the red point (ID 40543) and blue square (ID 40541). The calculated factor for above 300 T at the coil center is in the experimental range, which corresponds to 120 kA in the coil. This simulation result is consistent with the experimental result. The magnetization of relativistic electrons and magnetic mirror effect [9] account for the factor reduction shown in Supplementary Fig. 1 (c) with increasing applied magnetic field strength.

Supplementary Methods

Temporal change of magnetic field diffusion in a cone

Figure 3 of the main manuscript shows two-dimensional profile generated with a coil, in which 250 kA of current flows, only at the field peak timing. These profiles at three different timings are shown in Supplementary Fig. 2.

The upper, middle and lower figures were calculated with the constant electrical conductivities for $\sigma = 4 \times 10^7$ S m^{-1}, $\sigma = 2 \times 10^6$ S m^{-1} and calculated with consideration of temporal changes in temperature and conductivity of a gold cone due to inductive heating, respectively. Difference of diffusion process is clearly seen. More details of the model and scheme for this calculation is described in Ref. [10].

Supplementary References

[1] Santos, J. J. *et al.* Laser-driven platform for generation and characterization of strong quasi-static magnetic fields. *New J. Phys.* **17**, 083051 (2015).

[2] Law, K. F. F. *et al.* Direct measurement of kilo-tesla level magnetic field generated with laser-driven capacitor-coil target by proton deflectometry. *Appl. Phys. Lett.* **108**, 091104 (2016).

[3] Gao, L. *et al.* Ultrafast proton radiography of the magnetic fields generated by a laser-driven coil current. *Phys. Plasmas* **23**, 043106 (2016).

[4] Goyon, C. *et al.* Ultrafast probing of magnetic field growth inside a laser-driven solenoid. *Phys. Rev. E* **95**, 033208 (2017).

[5] Zhu, J. B. *et al.* Strong magnetic fields generated with a simple open-ended coil irradiated by high power laser pulses. *Appl. Phys. Lett*. **107**, 261903 (2015).

[6] Johzaki, T. Nakao, Y. and Mima, K. Fokker–Planck simulations for core heating in subignition cone-guiding fast ignition targets. *Phys. Plasmas* **16**, 062706 (2009).

[7] Nagatomo, H. *et al.* Compression and electron beam heating of solid target under the external magnetic field for fast ignition. *Nucl. Fusion* **57**, 086009 (2017).

[8] Arikawa, Y. *et al*. Improvement in the heating efficiency of fast ignition inertial confinement fusion through suppression of the preformed plasma. *Nucl. Fusion* **57**, 066022 (2017).

[9] Johzaki, T. *et al.* Control of an electron beam using strong magnetic field for efficient core heating in fast ignition. *Nucl. Fusion* **55**, 053022 (2015).

[10] Morita, H., Sunahara, A., Arikawa, Y., Azechi, H. & Fujioka S. Two-Dimensional Computation of Pulsed Magnetic Field Diffusion Dynamics in Gold Cone with Consideration of Inductive Heating and Temperature Dependence of Electrical Conductivity. Preprint at http://arxiv.org/abs/1804.10410 (2018).

Supplementary Figure 1

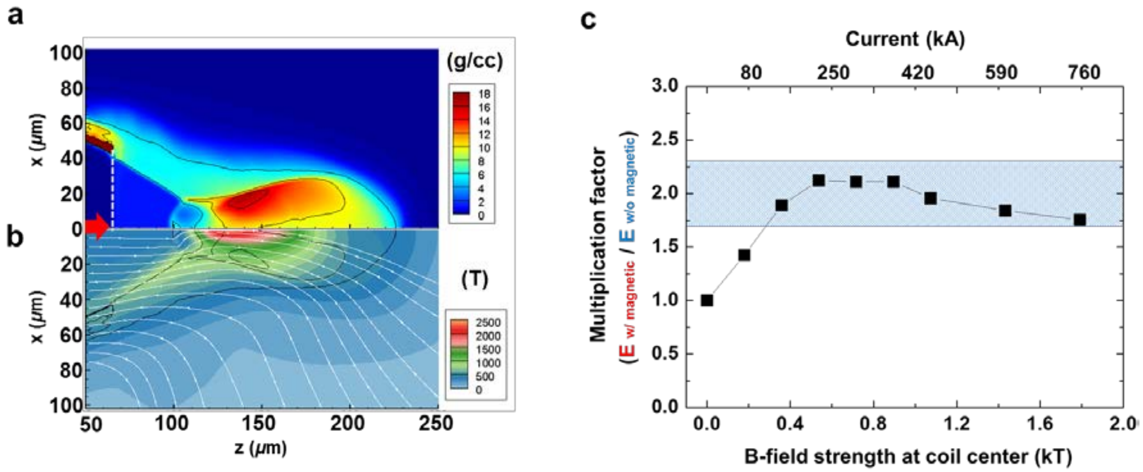

REB transport simulation for several imposed magnetic field strength.

Enhancement of REB-to-core energy coupling was calculated with the integrated simulation by coupling two-dimensional radiative MHD code and two-dimensional hybrid code. Spatial profiles of (a) density and (b) magnetic field at the maximum compression timing that were calculated with two-dimensional radiative MHD simulation code. (c) Dependence of multiplication factor of REB-to-core energy coupling on the initial magnetic field strength at the coil center.

Supplementary Figure 2

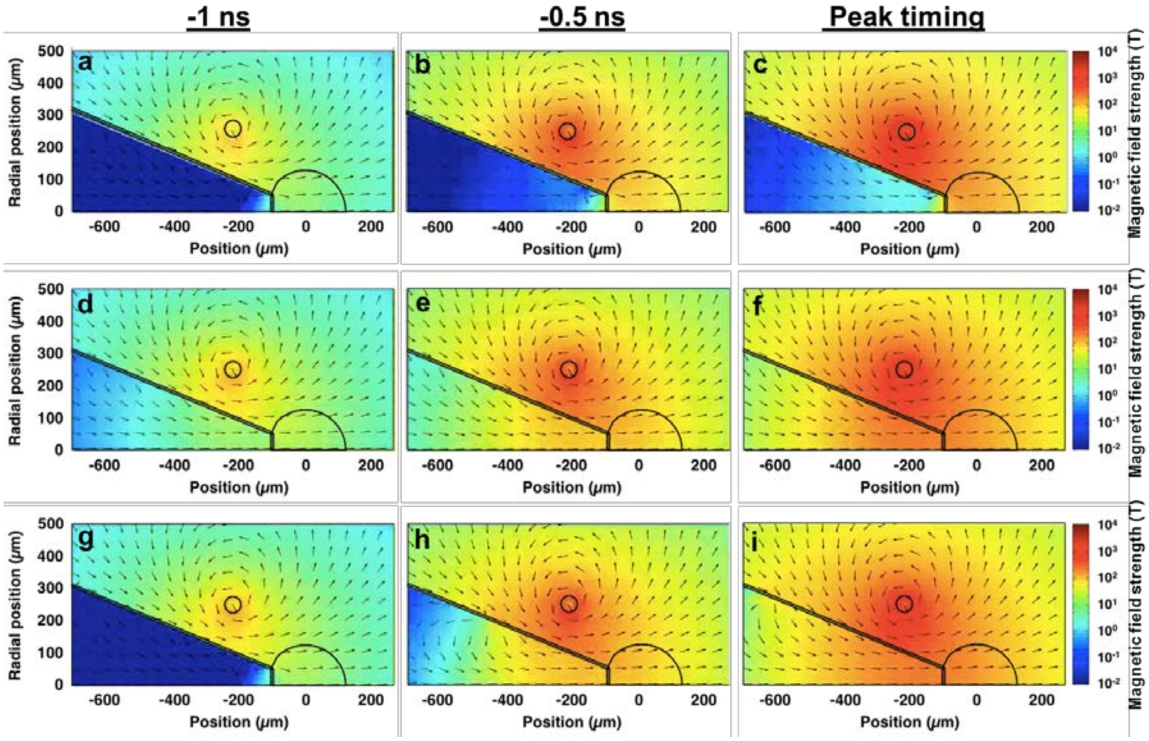

Two-dimensional profiles of the magnetic field calculated with different conductivities.

Two-dimensional profiles of the magnetic field at three different timings (-1 ns, -0.5 ns, peak timing) are calculated with the constant conductivity ($\sigma = 4 \times 10^7$ S m^{-1} for the (a, b, c) and $\sigma = 2 \times 10^6$ S m^{-1} for the (d, e, f)) and with consideration of temporal changes in temperature and conductivity of gold due to inductive heating (g, h, i).

Supplementary Table 1

Summary of magnetic field strength measured on kilo-Joule class laser facilities. Magnetic field strength with various laser energy, irradiance, pulse duration, coil shape and material were measured by some methods on large laser facilities.

Current flows in the coils were evaluated from the measured magnetic field strengths with resistances and inductances that were calculated for the initial coil geometries. 200 kA-level currents were obtained with except for Ref. [3].

Facility /Country	Laser Energy (J)	Irradiance ($W \cdot cm^{-2} \cdot \mu m^2$)	Pulse duration (ns) / shape	Coil diameter (mm) / Material	Methods	B-field strength @center (T)
LULI (France) [Ref.1]	500	1×10^{17}	1.0 / Square	0.5 / Ni	· Proton<br>· B-dot<br>· Faraday	**600 +/- 10 (300 kA)**
GEKKO-XII (Japan) [Ref.2]	540	2×10^{16}	1.3 / Gaussian	0.5 / Ni	· Proton<br>· B-dot	**610 +/- 30 (250 kA)**
OMEGA EP (U.S.) [Ref.3]	1250	2×10^{15}	1.0 / Square	0.3 / Cu w / spacer	· Proton	**50 (22 kA)**
OMEGA EP (U.S.) [Ref.4]	750	5×10^{14}	0.75 / Square	0.5 / Au	· Proton<br>· Faraday	**210 +/- 35 (180 kA)**
Shengguang-II (China) [Ref. 5]	1970	7×10^{14}	1.0 / Square	1.16 / Cu	· Proton<br>· B-dot	**205 (200 kA)**
